# Material‐to‐Application Integration: Rapid Fabrication of Field‐Deployable Hydrogel‐SiO_2_ DNA Separator for Low‐Resource Point‐of‐Care Diagnostics

**DOI:** 10.1002/advs.202508580

**Published:** 2025-08-28

**Authors:** Peipei Li, Xinrong Li, Haojie Wu, Dongmei Yue, Yubo Shang, Shan Gao, Bai Wang, Xiaobin Jiang, Jingwei Jiang, Zunchun Zhou

**Affiliations:** ^1^ Ministry of Agriculture and Rural Affairs Key Laboratory of Aquatic Germplasm Resources Conservation and Utilization Liaoning Ocean and Fisheries Science Research Institute Liaoning Academy of Agricultural Sciences Dalian Liaoning 116023 China; ^2^ State Key Laboratory of Fine Chemicals School of Chemical Engineering Dalian University of Technology Dalian Liaoning 116024 China; ^3^ Liaoning Key Laboratory of Germplasm Improvement and Fine Seed Breeding for Marine Aquatic Animals Dalian Liaoning 116023 China

**Keywords:** hydrogel fabrication, nucleic acid extraction, point‐of‐care detection, resource‐limited settings, surface modification

## Abstract

Current nucleic acid (NA) diagnostics are hindered in resource‐limited settings by equipment needs and high costs. S(PAA‐SiO_2_) is developed, a hydrogel‐SiO_2_ composite deoxyribonucleic acid (DNA) separator addressing these challenges through rapid (<10 min), simple, and skill‐free preparation, ultralow cost ($0.028 per unit), and equipment‐minimized mass production capacity (96 units per batch). Further systematic investigation of SiO_2_ particle surface modification revealed critical enhancements in DNA adsorption properties. With maintaining structural thermal stability, SiO_2_ integration significantly improved the surface roughness, specific surface area, and hydrophobicity, leading to hydrophobic and salt bridge effect‐enhanced DNA adsorption. Especially, SiO_2_ modification with mixed particle size formed a hierarchical microstructure to promote turbulence and interface interaction. Device S(PAA‐SiO_2_‐Mix) achieved a higher DNA extraction yield than the commercial kits. It possesses potential value for Polymerase Chain Reaction (PCR) diagnosis of bacterial, viral, parasitic, and fungal pathogens. By orchestrating three technological progresses ‐ rapid S(PAA‐SiO_2_‐Mix) fabrication, high‐throughput DNA extraction, and visual loop‐mediated isothermal amplification (LAMP) ‐ a field‐deployable diagnostic workflow is established for *Vibrio parahaemolyticus (v. parahaemolyticus, Vpa)*. This platform achieves equipment‐minimized point‐of‐care detection in <40 min with a sensitivity of 10 CFU mL^−1^. Its simplicity, speed, and accuracy offer a transformative solution for resource‐limited diagnostics. This work advances separation interface design and presents a novel vision for integrated, equipment‐free molecular detection systems.

## Graphical abstract

A rapidly fabricated hydrogel‐SiO_2_ composite enables ultralow‐cost, equipment‐minimized DNA extraction, outperforming commercial kits. Hierarchical microstructures enhance hydrophobic/salt‐bridge‐driven adsorption. Integrated with visual LAMP, this field‐deployable system detects *Vibrio parahaemolyticus* at 10 CFU mL^−1^ in <40 min, offering transformative point‐of‐care diagnostics for resource‐limited settings.

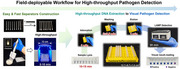

## 1 Introduction

Effective pathogen detection is critical for disease control, preventing transmission by unidentified carriers.^[^ ^1^ ^]^ NA‐based methods, particularly PCR,^[^ ^2^ ^]^ are primary diagnostic tools due to their adaptability, sensitivity, and speed. However, their accuracy and timeliness hinge critically on the initial step: rapid, efficient extraction of high‐quality NA.^[^ ^3^ ^]^ Inadequate preparation risks false negatives,^[^ ^4^ ^]^ potentially enabling pathogen spread with severe consequences, as seen in COVID‐19.^[^ ^5^ ^]^

While solid‐phase extraction (SPE) offers advantages over liquid‐phase methods (faster processing, reduced contamination),^[^ ^6^ ^]^ current SPE approaches face limitations. Traditional non‐magnetic methods (e.g., columns) require complex centrifugation, hindering throughput and confining use to labs. Magnetic SPE using nanoparticles avoids centrifugation^[^ ^7^ ^]^ but suffers from aggregation/sedimentation affecting stability^[^ ^8^ ^]^ or requires harsh DNA adsorption/desorption conditions.^[^ ^9^ ^]^ These constraints severely impede crucial PCR diagnostics in resource‐limited settings ‐ precisely where disease burdens are high and rapid, accessible testing is essential.^[^ ^10^ ^]^ Consequently, developing novel, rapid NA extraction technologies is therefore imperative to ensure diagnostic efficiency, accessibility, and cost‐effectiveness, especially for large‐scale surveys and low‐resource areas.^[^ ^11^ ^]^

Recent advancements aim to overcome these hurdles. Examples include microneedle patches for plant DNA,^[^ ^12^ ^]^ cellulose/filter paper strips,^[^ ^13^ ^]^ and three dimensional (3D)‐printed polyacrylic acid (PAA) devices,^[^ ^14^ ^]^ all minimizing centrifugation and labor. Materials science offers further promise: hydrogels,^[^ ^15^ ^]^ rich in functional groups and biocompatible,^[^ ^16^ ^]^ exhibit potential NA adsorption‐desorption capacity^[^ ^17^ ^]^ via diverse interactions (electrostatic,^[^ ^18^ ^]^ van der Waals forces,^[^ ^19^ ^]^ hydrogen bonds,^[^ ^8^ ^]^ π‐π stacking,^[^ ^20^ ^]^ etc.); Silica (SiO_2_) remains a stable, cost‐effective adsorbent.^[^ ^21^ ^]^ These provide viable paths to rapid, high‐quality NA isolation, vital for accurate, timely detection in low‐resource scenarios critical for global health.

Here, we report an innovative SiO_2_‐modified hydrogel DNA separator. Engineered for enhanced DNA adsorption capacity through optimized interface interactions, it enables simple batch production (96 units/batch) using a portable ultraviolet (UV) lamp and rapid, adjustable high‐throughput extraction (1‐96 samples simultaneously). Coupled with LAMP technology and colorimetric detection,^[^ ^22^ ^]^ we further established a field‐deployable point‐of‐care detection method for the foodborne pathogen *Vpa*. This integrated approach pioneers a novel pathway for deployable NA analysis in both clinical and field settings.

## 2 Results and Discussion

### 2.1 Rapid and Simple Fabrication of the Device

Hydrogel materials possess abundant surface functional groups and excellent biocompatibility, making them potentially effective adsorbents for DNA. Our previous studies demonstrated these hydrogels’ high adsorption properties and the interface microstructure for the separation process.^[^ ^14a^ ^]^ High‐precision 3D printing technologies integrate high‐throughput macroscopic separator structures with precise regulation of the microscopic interface of hydrogel carriers. However, the high cost of 3D printers and the specialized skills required for device fabrication on the machine^[^ ^23^ ^]^ pose significant challenges in low‐resource environments. To address this issue, we propose a simple, low‐equipment‐dependent method for fabricating hydrogel DNA extraction devices suitable for such settings.

Surface roughness is crucial for increasing surface area and NA binding sites. Particle loading is an effective strategy to increase interface roughness under low‐resource formation conditions. In this study, PAA was used as the base material, and SiO_2_ particles were doped into the polymer through a UV polymerization reaction to construct the NA separation device. The selection of SiO_2_ particle size was informed by our preliminary experimental results and hypotheses. Initially, nano‐sized SiO_2_ particles (25–50 nm) were employed; however, severe particle detachment occurred following polymerization. Considering the porous feature of the hydrogel and the exothermicity of the polymerization process, we propose that the polymerization predominantly forms a “particle‐hydrogel” topological 3D structure through mechanisms of entrapment and embedding. The hydrogel's pore size exceeded that of the nano‐SiO_2_ particles, resulting in particle detachment. Concurrently, the heat release during polymerization promoted nanoparticle diffusion, exacerbating surface detachment. Hence, micron‐sized SiO_2_ particles were selected for this study. To explicitly investigate the impact of micron‐scale particle size, we selected two distinct SiO_2_ particle size ranges (40–100 and 180–270 µm) commonly available commercially. These ranges exhibit no overlap and possess significantly different primary particle sizes. Furthermore, based on the theory that multimodal particle size distributions enhance turbulence and interfacial mass transfer in hierarchical structures,^[^ ^24^ ^]^ we implemented a mixed‐particle‐size SiO_2_ loading strategy.

**Figure 1:**
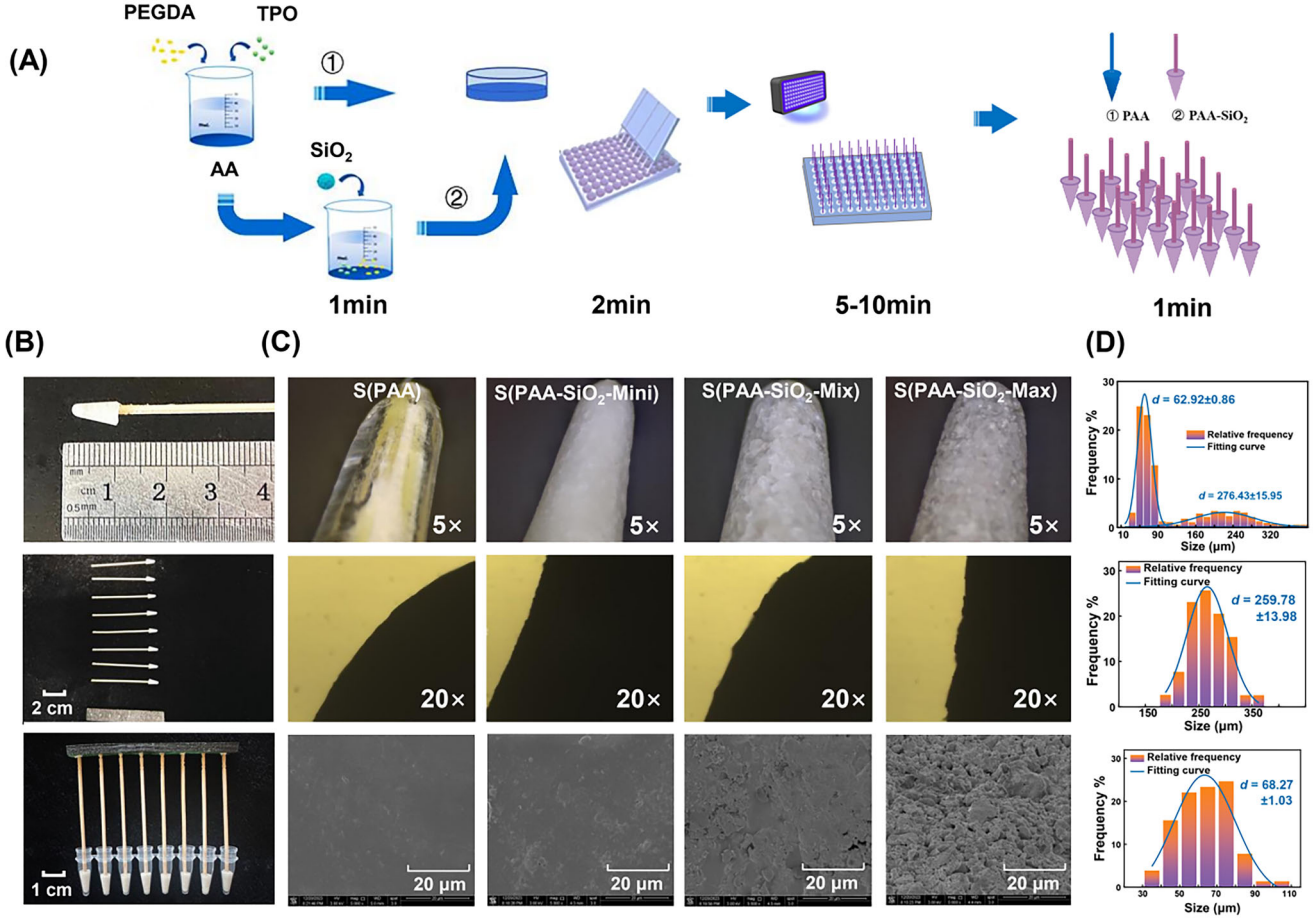
Fabrication Process and Characterization of NA Separation Devices. A) Diagram of the fabrication process. B) Images of a single and assembled device. C) Surface morphology by electron microscope. D) Particle size distribution analysis.

A 0.2 mL commercial centrifuge tube was served as mold. The height of functional part was set at 1 cm, approximately the height of the bottom conical part of the tube. This size will enable the device to be inserted into the bottom of most molecular biology consumables to the greatest extent: 1.5/2.0 mL microcentrifuge tubes and 8‐tube strips or 96‐well plates (0.2 mL) for high‐throughput operations. The inexpensive toothpicks (or any rod‐shaped material) acted as handles, while lightweight, quick‐drying clay was used to connect individual units into an integrated device compatible with 8‐well or 96‐well plates. The design of the device allows for adjustable mold sizes, handle lengths, and connection configurations, thereby enhancing its applicability across various biomedical scenarios (as shown in **Figure** **1**).

Following UV irradiation and curing, the device can be easily demolded by pulling it outward using the handle. To meet the requirements of subsequent PCR tests, commercial 0.2 mL 96‐well plates were used as molds to fabricate devices in batches, and eight devices were connected with strips made from lightweight clay. The hydrogel functional part is 10 mm in height and is specifically shaped to fit within the centrifuge tube (Figure 1B).

Notably, the entire fabrication process requires no laboratory power equipment and is accomplished with only a mini UV lamp (dimensions: 15 cm × 8 cm × 6 cm). This streamlined approach enables the batch production of NA extraction devices, yielding 96 or 384 units per batch. The fabrication process is rapid, typically taking 5–10 min, depending on mold size, and does not necessitate specialized skills. The cost of production is minimal, with each S(PAA‐SiO_2_) device costing under $0.028 and each S(PAA) device costing $0.023. In conclusion, this fabrication method for NA functional devices is particularly well‐suited for low‐resource environments and underdeveloped areas.

### 2.2 Characterization and Analysis

We characterized the obtained separation devices. For convenience, we denote the devices without SiO_2_ modification as S(PAA) and those with SiO_2_ as S(PAA‐SiO_2_). Devices were further classified by SiO_2_ particle size: S(PAA‐SiO_2_‐Mini) for small particles (40–100 µm), S(PAA‐SiO_2_‐Max) for large particles (180–270 µm), and S(PAA‐SiO_2_‐Mix) for a mix of both sizes (1:1 ratio).

High‐magnification microscopy analysis revealed distinct surface structures of S(PAA) and S(PAA‐SiO_2_). S(PAA‐SiO_2_) exhibited pronounced protrusions from SiO_2_ modification (Figure 1C), enhancing the specific surface area and NA binding sites. High‐magnification microscope images, particle size statistics, and Gaussian fitting characterization (shown in Figure 1D) revealed that S(PAA‐SiO_2_‐Mix) largely consists of smaller SiO_2_ particles (mainly at 62.92 µm, 26%), with a minor fraction of larger ones (at 276.45 µm, 5%). It might be related to the differentiated dispersion. As particle size increases, the ability to counteract sedimentation via thermal motion diminishes, leading to a significant increase in sedimentation velocity. In practical experimental operations, these differences are further amplified. During mixing/stirring, smaller particles are more readily captured and dispersed by fluid shear forces, while larger particles, due to their greater inertia, exhibit fluid avoidance behavior around the stirrer, leading to poorer dispersion of large size SiO_2_. The particle sizes in S(PAA‐SiO_2_‐Mini) and S(PAA‐SiO_2_‐Max) correspond to the intended particle size ranges for SiO_2_ modification. S(PAA‐SiO_2_‐Max) predominantly had particles at 256.78 µm (25%), while S(PAA‐SiO_2_‐Mini) featured sizes ≈68.27 µm (25%).

**Figure 2:**
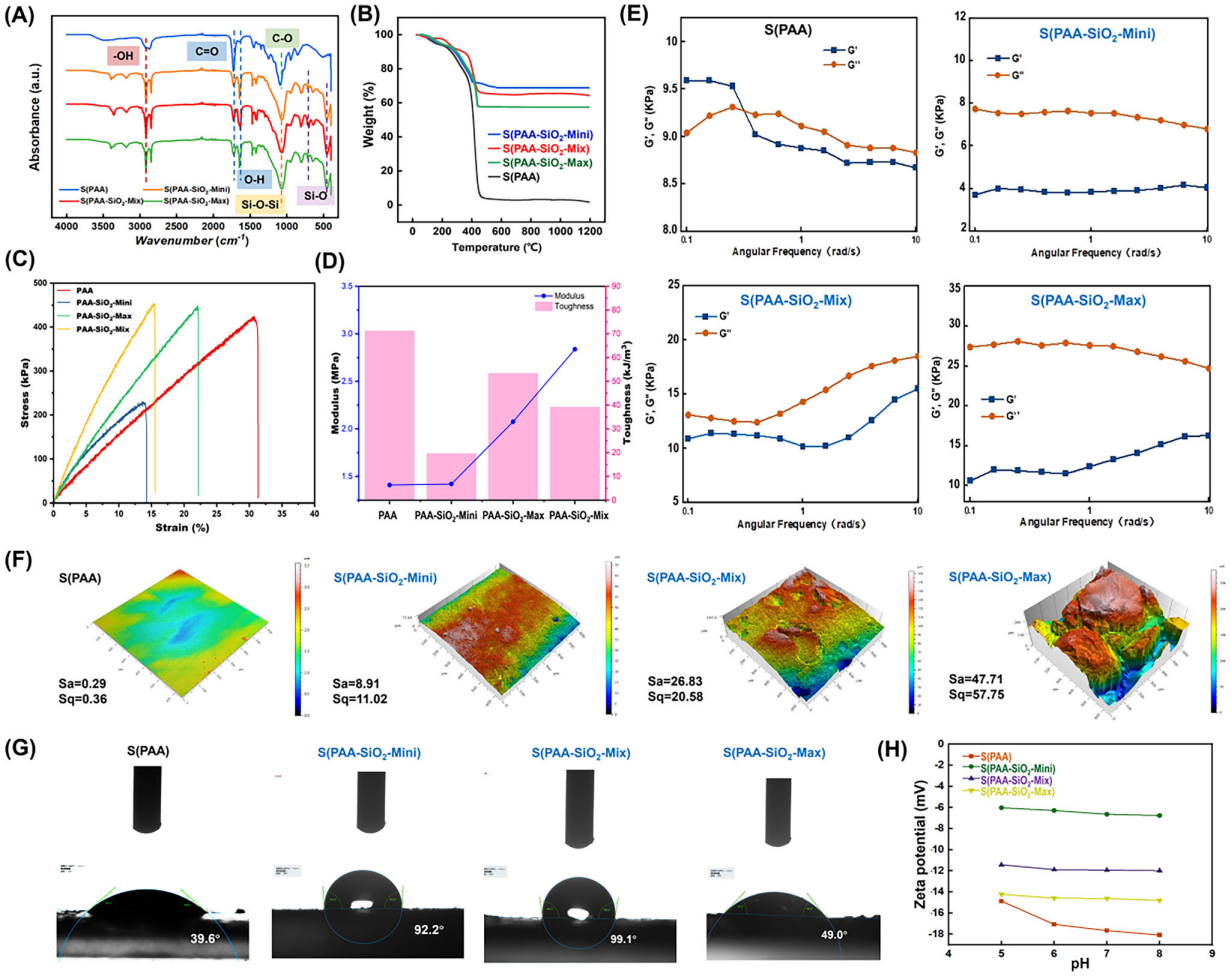
Characterization of the DNA Separation Devices. A) FT‐IR. B) TGA. C) Stress‐strain curve D) Modulus‐toughness curve. E) Viscous and elastic response. F) Surface 3D confocal laser microscopy characterization. G) CA. H) Zeta potential.

FTIR analysis confirms the successful loading of SiO_2_. As shown in **Figure** **2**A, the fourier transform infrared spectrum (FT‐IR) of S(PAA) features a strong absorption peak at 1160 cm^−1^, indicating the stretching vibration of the ester C─O group in the cross‐linking agent polyethylene glycol diacrylate (PEGDA). The S(PAA‐SiO_2_) device shows a strong absorption peak at 1110 cm^−1^, which corresponds to the asymmetric stretching vibration of the Si─O─Si bond, and the ester C─O stretching vibration of the cross‐linking agent, forming a broad peak. Additional absorption peaks at 795 and 469 cm^−1^ relate to symmetric stretching vibrations of Si─O, while the 1630 cm^−1^ peak corresponds to the bending vibration of O─H. Peaks at 1716 and 3000 cm^−1^ are attributed to the stretching vibrations of C═O and ─OH, respectively. A broad peak at 3450 cm^−1^ indicates the asymmetric stretching vibration of structural water (O─H), and the peak near 1638 cm^−1^ is associated with the bending vibration of H‐O‐H in water. Other peaks, including the C─H stretching at 2920 cm^−1^ and bending vibrations for ‐CH_3_ and C‐H at 1340 and 1470 cm^−1^, are due to cross‐linking agent initiators. The C═O peak (1716 cm^−1^) in S(PAA‐SiO_2_) is diminished, likely due to a reduction in PAA content as SiO_2_ occupies part of the material's volume.

Then, thermogravimetric analysis (TGA) was conducted to evaluate the thermostability of the functional 3D devices ^[^ ^25^ ^]^ As illustrated in Figure 2B, both S(PAA) and S(PAA‐SiO_2_) exhibit stability up to 100 °C, indicating ease of storage and application under varying conditions. At 100 °C, water and solvents evaporate, while degradation of acrylic acid alcohol, urethane bonds, and other structures occurs between 100 °C and 400 °C under nitrogen. PAA undergoes complete decomposition ≈480 °C, and when heated to above 1000 °C, the residual materials of S(PAA‐SiO_2_‐Mix), S(PAA‐SiO_2_‐Mini), and S(PAA‐SiO_2_‐Max) are exclusively composed of SiO_2_. Overall, the modification of SiO_2_ does not compromise thermal stability, ensuring that physical properties remain stable during NA extraction, which is typically performed at room temperature.

The mechanical properties of the materials were also characterized. As shown in Figure 2C,D, PAA exhibited a fracture strain of 31.32%, a fracture strength of 425.11 kPa, and a tensile modulus of 1.41 MPa. Upon incorporation of mini‐SiO_2_ particles, both the fracture strain and fracture strength decreased (14.22% and 174.00 kPa, respectively). This reduction is probably attributed to the extremely high surface energy of small‐diameter SiO_2_ particles, which promotes agglomeration. Within the hydrogel network, agglomerated small SiO_2_ particles form hydrogen bonds with polymer chains, leading to significant stress concentration during stretching. Consequently, both fracture strain and strength decrease, failing to enhance the hydrogel's mechanical performance. In contrast, adding max‐SiO_2_ particles resulted in a slight decrease in fracture strain to 22.17%, while the fracture strength remained largely unchanged at 446.08 kPa. However, the elastic modulus increased to 2.08 MPa. The low surface energy of large‐diameter SiO_2_ particles facilitates their supportiveness within the PAA hydrogel network without forming strong interactions with polymer chains, thus minimally affecting fracture strength. Nevertheless, the dispersed large particles impede the sliding of PAA chains during stretching, increasing the hydrogel's stiffness and thereby elevating the elastic modulus, albeit at the expense of reduced fracture strain. When using mix‐SiO_2_, the hydrogel demonstrated a fracture strain of 15.34%, a fracture strength of 453.25 kPa, and an elastic modulus of 2.84 MPa. This behavior arises from a synergistic effect between the differently sized SiO_2_ particles. Small particles enhance interfacial interactions, while large particles provide filling effects and structural support. This synergy improves the fracture strength to some extent. Moreover, the presence of large particles disperses the stress concentration induced by small particles, partially mitigating the negative effects associated with the smaller size. Additionally, the mixed particle sizes promote the formation of a multiscale structure within the hydrogel, facilitating efficient stress transfer and distribution. Small particles fill voids between larger particles, enhancing local strength, while the combined particle sizes enable more effective stress transmission and dispersion. Under external load, large particles initially bear part of the stress, which is then transferred via interfaces to small particles and ultimately dissipated throughout the matrix, preventing excessive stress concentration.

Viscoelastic analysis (Figure 2E) revealed that S(PAA) exhibits dominant elastic behavior (storage modulus G′ > loss modulus G″) at low angular frequencies. As frequency increases, a viscoelastic transition (G′ = G″) occurs, and viscous behavior gradually dominates, indicating irreversible deformation of the internal network structure. In contrast, S(PAA‐SiO_2_) primarily exhibits viscous response (G″ > G′) across the tested frequency range, with reduced elastic contribution. Particle size significantly influences the modulus of hydrogel composites. Mini‐SiO_2_, due to their high surface energy, may interfere with hydrogel polymerization. The resulting modulus reduction from incomplete polymerization outweighs any reinforcing effect, leading to a net decrease in modulus compared to S(PAA). Agglomeration of small particles, forming loose structures, further exacerbates this effect. For large particles, the reinforcing effect dominates, increasing both G2 and G3 due to their superior load‐bearing capacity and structural energy dissipation. Furthermore, SiO_2_ particles occupy sites otherwise filled by polymer, reducing polymer content per unit volume. This reduction causes fluctuations in the material's response at high frequencies. When using mix‐SiO_2_ particles, the gradation effect optimizes inter‐particle packing. This facilitates a gradual transition in the matrix behavior from viscoelastic toward more elastic‐like characteristics.

The interface “adsorption–desorption” performance is crucial for the DNA extraction process. We assessed the interface roughness, hydrophobicity, and charge properties of the constructed devices. As demonstrated by 3D confocal laser microscopy (Figure 2F), the surface of S(PAA) is flat (Sa = 0.291). The modification of SiO_2_ particles significantly enhanced interface roughness, which increased with larger particle sizes. S(PAA‐SiO_2_‐Mini) exhibits minimally increased surface roughness (Sa = 8.91). Of S(PAA‐SiO_2_‐Max), large particle size SiO_2_ is semi‐embedded and semi‐exposed in the PAA material, leading to the most significant increase in interface roughness (Sa = 47.71). The roughness improvement in S(PAA‐SiO_2_‐Mix) (Sa = 26.83) is between that of S(PAA‐SiO_2_‐Mini) and S(PAA‐SiO_2_‐Max).

The contact angle (CA) serves as an indicator of hydrophobicity or hydrophilicity. As shown in Figure 2G, the CAs of S(PAA‐SiO_2_‐Mini) (92.2°) and S(PAA‐SiO_2_‐Mix) (99.1°) were similar and significantly higher than those of S(PAA) (36.6°) and S(PAA‐SiO_2_‐Max) (49.0°), indicating the increased hydrophobicity. PAA, being a hydrophilic polymer, exhibits a 3D network with numerous hydrophilic groups, resulting in high surface energy and a small contact angle. The modification of SiO_2_ particles in the polymer reduced the density of hydrophilic groups of PAA, thus lowering the hydrophilicity, while simultaneously increasing the contact angle due to enhanced roughness. Surface modification of mix‐SiO_2_ demonstrated a more pronounced enhancement effect. Additional measurements of five S(PAA‐SiO_2_‐Mix) fabricated in different batches showed high consistency, with values ranging from 98.3° to 101.42° (mean = 99.8° ± 1.62°) (Figure S1, Supporting Information). Notably, despite S(PAA‐SiO_2_‐Max) having the highest roughness, the large and irregular SiO_2_ particles are partially exposed on the surface, creating an overly uneven surface that affects the surface tension of the liquid droplet. As a result, its contact angle is smaller than that of S(PAA‐SiO_2_‐Mix) and S(PAA‐SiO_2_‐Mini).

The Zeta potential can reflect the charge properties of the interface. We tested the Zeta potential of different DNA separation devices under varying pH levels (Figure 2H). The S(PAA) device exhibited a Zeta potential ranging from −14.90 to −18.07 mV at pH 5.0 to 8.0, indicating strong electronegativity on its surface. In contrast, the Zeta potential of the three S(PAA‐SiO_2_) devices decreased under the same conditions, with average values of −6.40, −11.79, and −14.53 mV for PAA‐SiO_2_‐Mini, PAA‐SiO_2_‐Mix, and PAA‐SiO_2_‐Max, respectively. These results suggest that the modification of SiO_2_ effectively reduces the surface electronegativity of the devices, with the following electronegativity trend: S(PAA‐SiO_2_‐Mini) < S(PAA‐SiO_2_‐Mix) < S(PAA‐SiO_2_‐Max) < S(PAA). As observed in our experiments, the reduction in electronegativity is conducive to the adsorption of negatively charged DNA molecules,^[^ ^26^ ^]^ particularly at pH 8. Notably, the surface electronegativity of the S(PAA‐SiO_2_) devices exhibited minimal variation with changes in pH, in contrast to S(PAA). This may be attributed to the dipole and electrostatic interactions between the hydroxyl groups on SiO_2_ and the carboxyl groups in PAA, which are polar functional groups. Furthermore, the stable chemical nature of SiO_2_ particles, the absence of ionizable species on their surface, and their resistance to environmental changes likely contribute to the high stability of the Zeta potential on the S(PAA‐SiO_2_) surfaces during testing.^[^ ^27^ ^]^

### 2.3 Nucleic Acid Separation Performance of the Device

#### 2.3.1 DNA Separation Process

**Figure 3:**
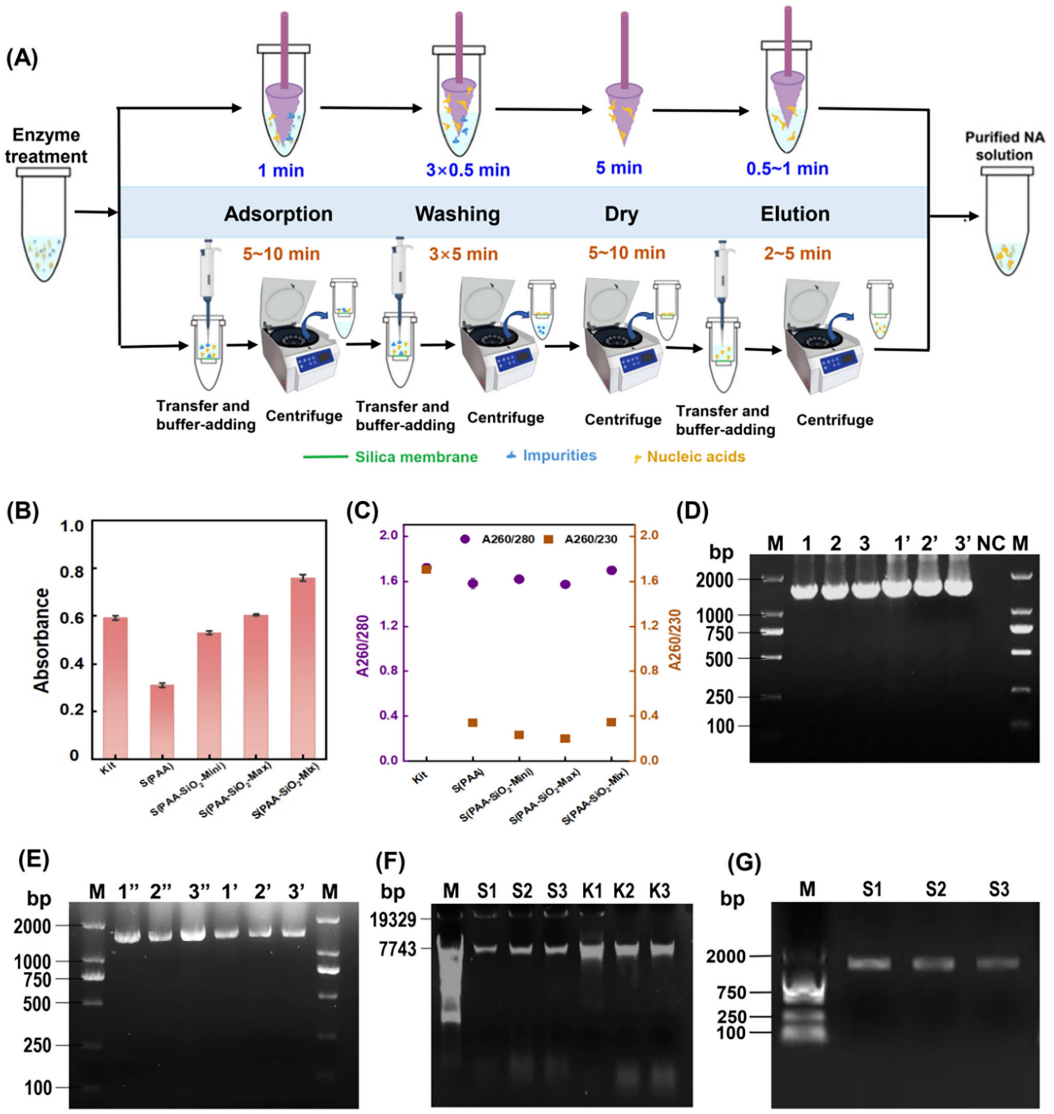
Evaluation of DNA extraction performance. A) Schematic of the extraction process using separators and a commercial Kit. B) A260 absorbance of extracted DNA. C) A260/280 and A260/230 ratios. D,E) Gel electrophoresis of PCR products amplified from bacterial DNA extracted via different extraction methods. Three biological replicates were shown for each method: DNA extraction via commercial kit (Lanes 1‐3), S(PAA‐SiO_2_‐Mix) (lanes 1′‐3′), and S(PAA) (lanes 1′’‐3′’), M: DL2000 DNA Marker. F) Gel electrophoresis analysis of DNA extraction products. M: Marker‐λ‐*Eco*T14. DNA extraction via S(PAA‐SiO2‐Mix) (lanes S1‐S3) and commerial kit (lanes K1‐K3) G) Gel electrophoresis of recovered DNA fragments by S(PAA‐SiO_2_‐Mix), M: DL2000 DNA Marker.

The S(PAA) and S(PAA‐SiO_2_) devices developed in this study represent a novel, non‐centrifugal method for DNA separation. The schematic representation of the DNA extraction process is shown in **Figure** **3**A. For performance comparison, we evaluated a commercially available, column‐based extraction kit, a mainstream method for DNA extraction. The column‐based approach relies on centrifuge‐assisted separation, wherein the lysate, wash buffer, and elution buffer pass through a silica‐based column under centrifugal force to achieve DNA separation. However, this method requires multiple centrifugations (> 5 times for each sample) and liquid transfer rounds, making the process more complex and time‐consuming (≈30–40 min). In contrast, the non‐centrifugal DNA separation devices used in this study eliminate the need for centrifugation equipment and complex procedural steps. By simple mechanical transfer of the separators in different functional buffers, DNA separation is completed in less than 10 min, reducing the operation time to one‐quarter of that required by the column‐based method. The flexible and adjustable structure of the PAA hydrogel material makes it easy to construct specific structures that can be adapted to different separation conditions,^[^ ^16b^ ^]^ enabling controllable and high‐throughput DNA extraction. This method thus holds significant potential for rapid and simple DNA separation in emergency pathogen investigations or large‐scale sample processing, particularly in low‐resource settings.

#### 2.3.2 Nucleic Acid Separation Yield and Quality

Furthermore, we compared the performance of the devices and commercial kits in DNA extraction from *Vpa*, a gram‐negative bacterium isolated from raw seafood. The yield and quality of the extracted DNAs were evaluated and compared. The results showed that the interfacial modification of SiO_2_ particles significantly improved the DNA extraction yield of the devices. As shown in Figure 3B, the S(PAA‐SiO_2_‐Mix) device generally demonstrated the highest DNA extraction efficiency, with an average yield of 37.97 ng µL^−1^ (in terms of double‐stranded DNA, dsDNA), significantly higher than the other devices and yielding 1.28 times more DNA than the commercial kit (29.63 ng µL^−1^ dsDNA). S(PAA‐SiO_2_‐Max) (30.23 ng µL^−1^, dsDNA) and S(PAA‐SiO_2_‐Mini) (26.51 ng µL^−1^, dsDNA) devices showed comparable yields to the commercial kit, while S(PAA) displayed a relatively low yield (15.53 ng µL^−1^ dsDNA). However, the effect of SiO_2_ modification on DNA extraction quality was minimal, with A280/260 ratios similar to the commercial kit (Figure 3C). It indicates a low risk of protein contamination and demonstrate high DNA adsorption selectivity for both the commercial kit and devices. While A260/230 ratios for S(PAA) and S(PAA‐SiO_2_) extracted DNAs were slightly lower, this neither reflects compromised DNA purity nor impacts their suitability for PCR diagnostics. As shown in Figure 3D,E, amplifications using DNA from both devices achieved performance comparable to the commercial kit, with no PCR inhibition observed. Successful target gene amplification constitutes the essential prerequisite for biological applications. The observed decrease in A260/230 values may be attributed to the salt bridge‐mediated DNA adsorption mechanism, which may retain elevated salt ion concentrations. This mechanism will be comprehensively analyzed in the subsequent discussion.

Additionally, the integrity of the extracted DNA was verified by gel electrophoresis. As shown in Figure 3F, DNA extracted from *Vpa* using both the S(PAA‐SiO_2_) device and the commercial kit exhibited good integrity. The DNA band observed within the 7000–8000 bp range corresponds to a smaller plasmid (7642 bp) in the *Vpa* genome.^[^ ^28^ ^]^ In contrast, the two chromosomes (3416467 and 1843316 bp) and the two larger plasmids (92495 and 83481 bp) of *Vpa* were not readily visualized by agarose gel electrophoresis.

Given that the *Vpa* plasmid is circular, these results indicate that the functional device is suitable for extracting circular DNA. Furthermore, to verify the device's efficacy in adsorbing linear DNA, we processed a PCR product (a linear DNA fragment of ≈1800 bp) through device adsorption and elution, followed by gel electrophoresis. The electrophoresis result showed a single, intact band (Figure 3G), demonstrating the broad applicability of the separation device for both circular and linear DNA forms.

#### 2.3.3 Elution Efficiency and Reusability of the Device

**Figure 4:**
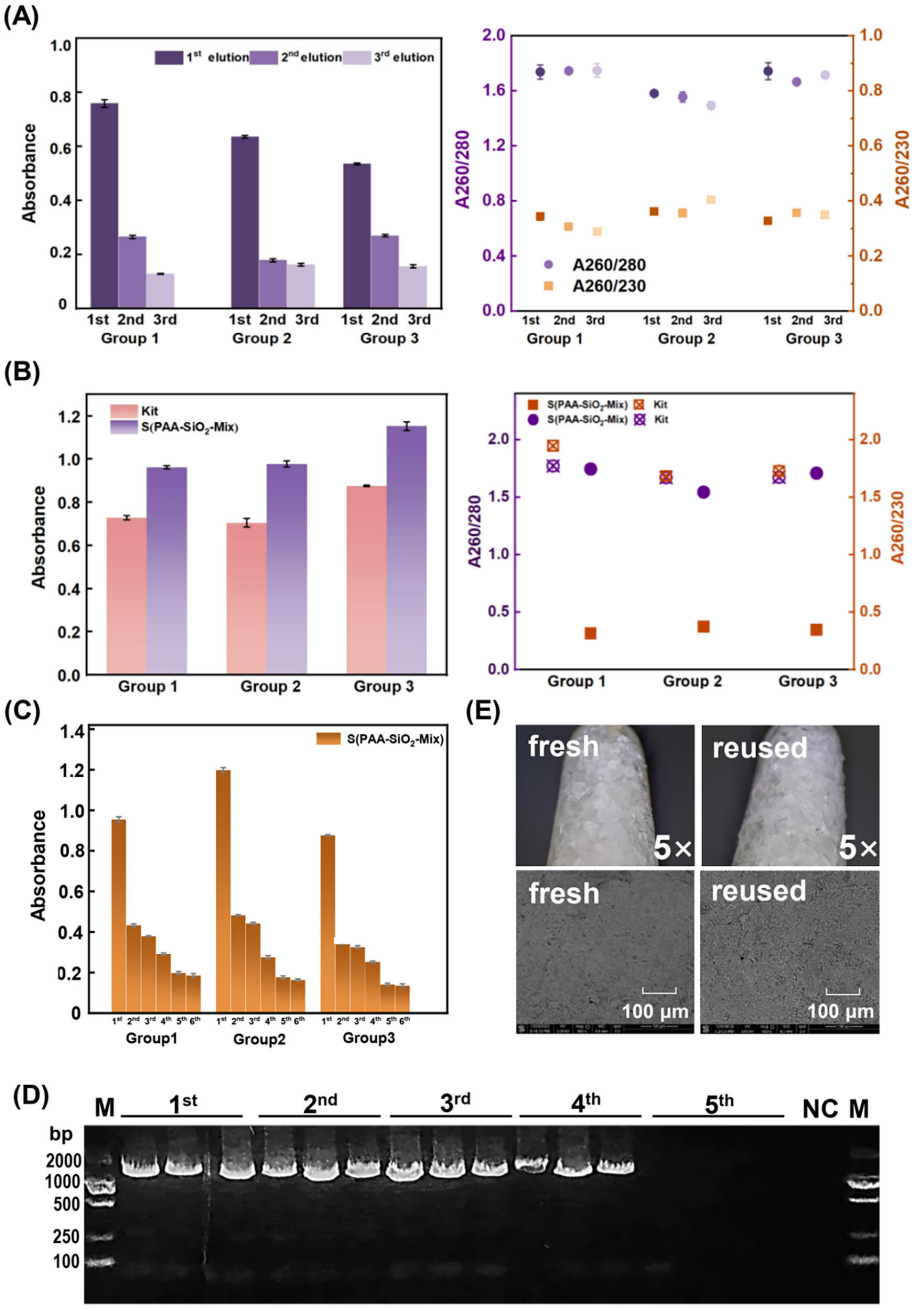
Evaluation of DNA separation performance using S(PAA‐SiO_2_‐Mix). A) A260 absorbance and A260/280 and A260/230 ratios for three elution. B) Comparison of A260 absorbance and A260/280 and A260/230 ratios of DNA extraction products using S(PAA‐SiO_2_‐Mix) versus a commercial Kit. C) A260 absorbance of sequential eluates (1st to 5th) from the DNA‐adsorbed S(PAA‐SiO_2_‐Mix). D) Gel electrophoresis of PCR products amplified from bacterial DNA in the sequential eluates. M: DL2000 Marker. E) Surface microscopy analysis of reused S(PAA‐SiO_2_‐Mix).

The DNA elution efficiency and reusability of the devices were systematically evaluated. Experimental results demonstrated that these devices exhibit advantages in simplified elution procedures and cost‐effective recyclability. To optimize DNA recovery, common protocols typically employ multiple elution steps, conventionally 2‐3 repetitions. In our study, the cumulative DNA yield from three sequential elutions was quantified as the total extraction yield. Comparative analysis revealed that the first‐elution efficiency of device S(PAA‐SiO_2_‐Mix) reached 66.32%, significantly surpassing the commercial kit's first‐elution efficiency of 53.61% (**Figure** **4**A,B). This enhanced single‐recycling rate positions the device as a competitive solution for rapid DNA extraction applications.

Quantitative evaluation showed that device S(PAA‐SiO_2_‐Mix) achieved total DNA yields ranging from 48.20 to 58.43 ng µL^−1^, outperforming the commercial kit's yield range of 36.40‐51.15 ng µL^−1^. These findings confirm that iterative elution effectively enhances total DNA recovery.

Post‐extraction recycling studies demonstrated that devices can be successfully reused following sequential aqueous washing cycles. Residual DNA analysis via PCR amplification showed progressive reduction in wash solution, with complete elimination observed after five washing cycles (Figure 4C,D). Structural integrity assessment through three consecutive “adsorption‐washing‐desorption” cycles revealed no significant surface degradation or mechanical defects (Figure 4E), confirming the device's durability and economic viability for repeated use.

### 2.4 DNA Separation Mechanism

The hybridization of SiO_2_ confers multifunctional properties encompassing intrinsic strength enhancement, surface roughening, and increasing DNA adsorption sites. The solid‐phase DNA separation process involves multimodal adsorption of DNAs to the adsorbent surface. Under aqueous conditions at pH 2.0‐9.0, DNA molecules carry negative charges, while S(PAA) possesses abundant carboxyl groups that deprotonate to exhibit negative charges in solution.^[^ ^29^ ^]^ Surface hydration of S(PAA‐SiO_2_) generates negatively charged silica hydroxyl groups, as confirmed by zeta potential measurements. Consequently, direct electrostatic attraction between DNAs and the negatively charged adsorbent surface is improbable. However, reduction of the adsorbent's negative charge density enhances DNA adsorption capacity. Our experimental data demonstrate that SiO_2_ hybridization effectively reduces the surface charge of the device, as evidenced by the decrease in zeta potential from ‐18.07 to ‐14.75 mV, resulting in superior DNA extraction yield for S(PAA‐SiO_2_) compared to S(PAA).

**Figure 5:**
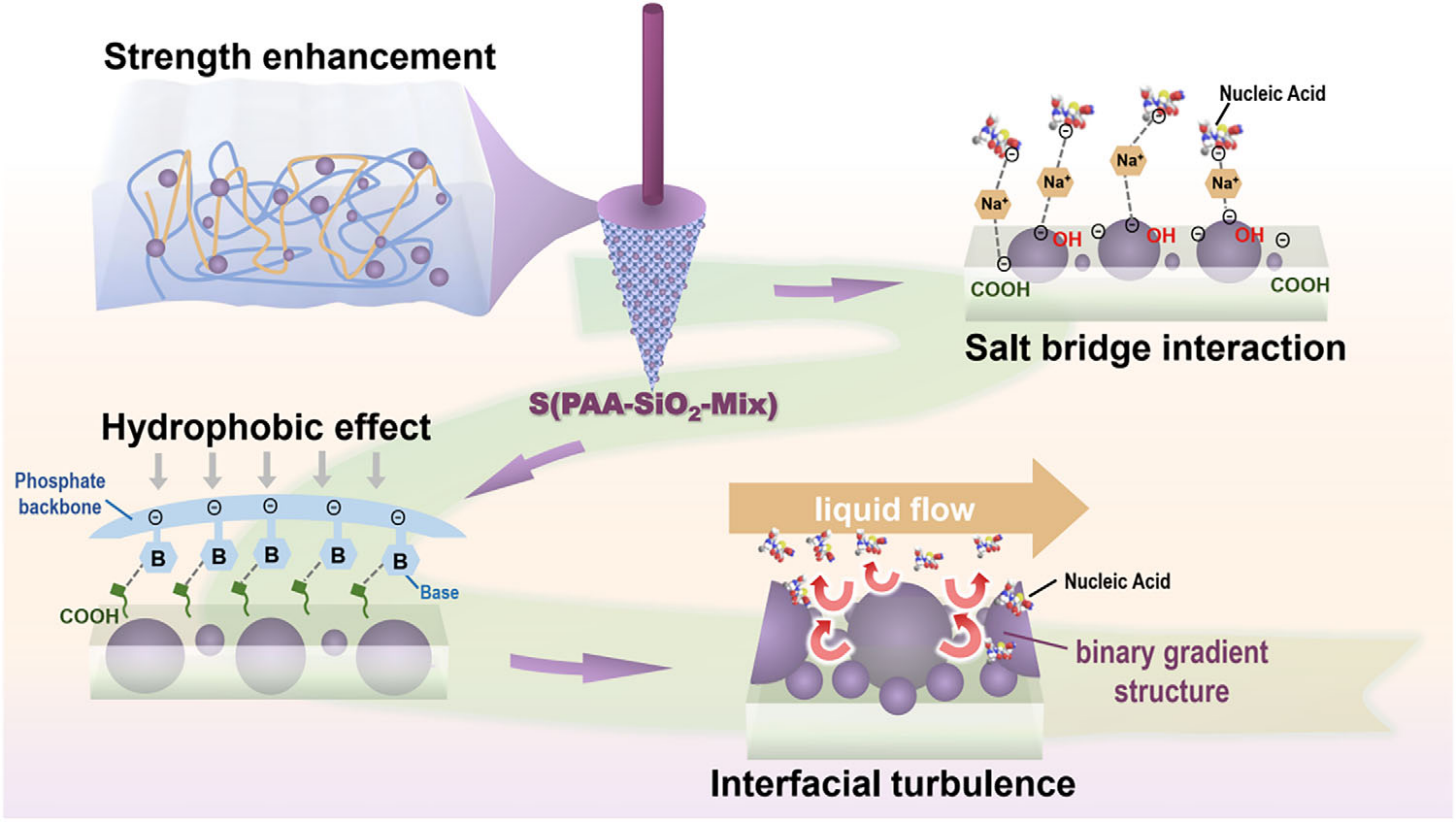
DNA separation mechanism of S(PAA‐SiO_2_‐Mix).

In this study, we argue that DNA adsorption to both S(PAA) and S(PAA‐SiO_2_) primarily occurs through salt bridge interactions and the hydrophobic effect.^[^ ^30^ ^]^ Within the lysis buffer system, the abundant Na^+^ ions accumulate around the negatively charged NA backbone and the negatively charged device surface to form an “ion cloud”, partially neutralizing the surface charges of both and thereby reducing the electrostatic repulsion.^[^ ^26^ ^]^ On the other hand, they act as counterions and facilitate the formation of an “anion‐cation‐anion” salt bridge structure, enabling specific adsorption of DNA molecules onto the device surface.^[^ ^31^ ^]^ The addition of isopropanol as a hydrophobic solvent further enhances the adsorption process. Given the insolubility of DNAs in isopropanol, their molecules preferentially separate from the aqueous phase and bind to the hydrophobic functional surface of the device via forces such as van der Waals interactions.^[^ ^32^ ^]^ Surface characterization through contact angle measurements and 3D confocal laser microscopy demonstrates that SiO_2_ loading significantly enhances the functional device's hydrophobicity and surface roughness. The increased surface roughness provides additional binding sites for salt bridge interactions, while the enhanced hydrophobicity promotes stronger hydrophobic interactions. Notably, the incorporation of various silica particle sizes within the material network not only improves mechanical strength but also creates a binary gradient interface structure. Although S(PAA‐SiO_2_‐Mix) does not exhibit the highest surface roughness, it demonstrates optimal hydrophobicity and overall performance, achieving the highest extraction yield. This enhanced performance can be attributed to the binary gradient structure, which increases interfacial turbulence between the device and lysis buffer, thereby promoting DNA‐device interactions and improving binding efficiency^[^ ^17^, ^24^ ^]^ (**Figure** **5**).

Considering the accessibility and universality of downstream NA applications, the most common low‐salt Tris‐EDTA (TE) buffer (pH = 8.0) was employed for DNA elution. The rapid reduction in cation concentration enhance the electrostatic repulsion between the NA molecules and the negatively charged device surface, weakening adsorption and simultaneously diminishing the ionic bridge effect, thereby enabling desorption.

### 2.5 DNA Extraction and Pathogen PCR Diagnosis

As demonstrated in our previous results, the S(PAA‐SiO_2_‐Mix) device exhibits superior DNA extraction performance, offering both high efficiency and equipment‐free operation, making it particularly suitable for low‐resource settings, especially for point‐of‐care and field clinical applications. The integration of this device‐based DNA extraction with a low‐equipment‐dependent DNA amplification technique enables rapid gene detection within a short timeframe, facilitating pathogen detection.

**Figure 6:**
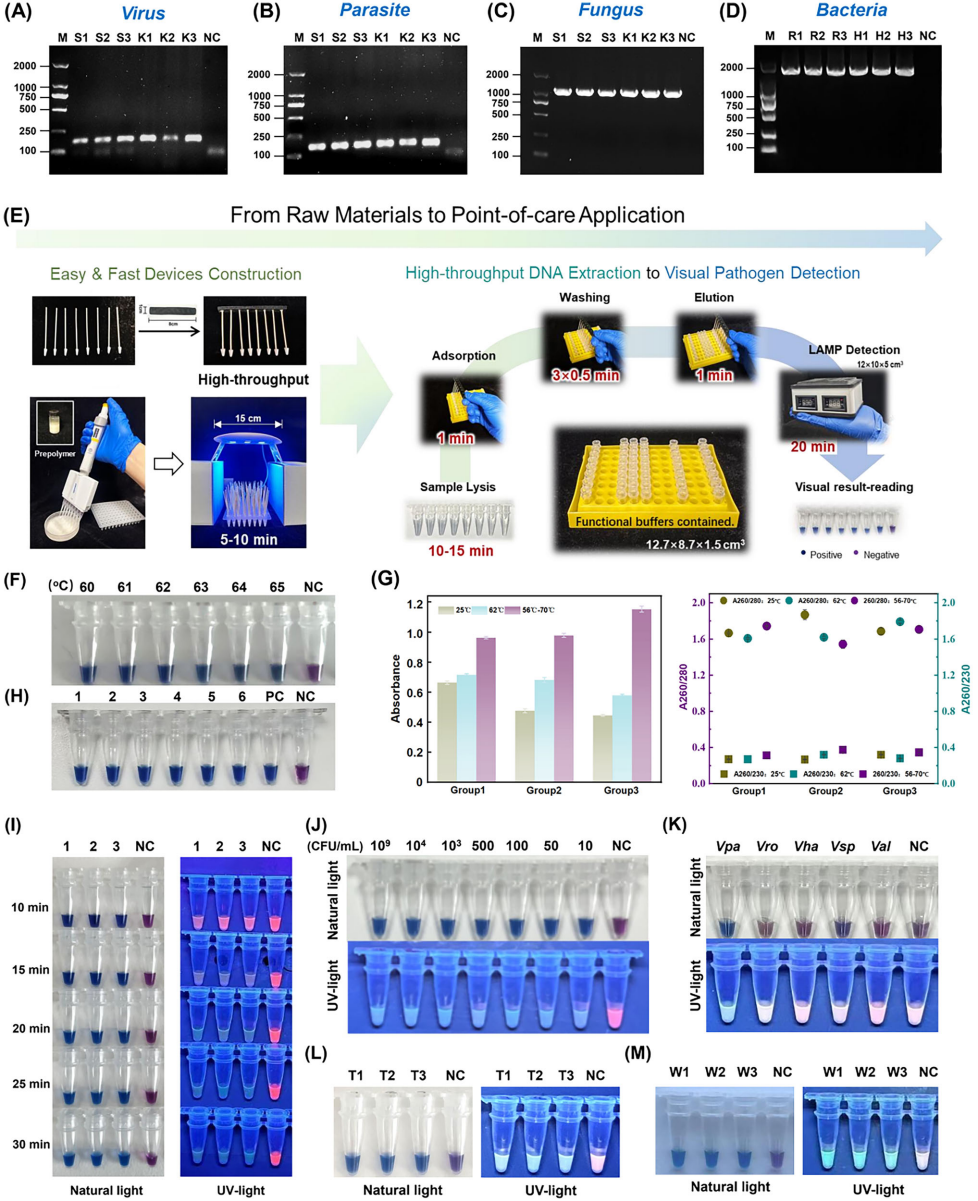
DNA extraction and pathogen diagnosis in low‐resource settings. A–C) Gel electrophoresis of PCR diagnosis of virus *NPV*, parasite *Np*, and a fungus. Lanes S1‐3: DNA templates prepared by S(PAA‐SiO_2_‐Mix); Lanes K1‐3: DNA templates prepared via commercial kits; M‐DL2000 DNA marker. D) Gel electrophoresis of PCR diagnosis of bacteria *Vpa* using DNA templates prepared by S(PAA‐SiO_2_‐Mix). Lanes R1‐3: three samples lysis under room temperature; H1‐3: three samples lysis under 62 °C. E) Technical process schematic diagram of S(PAA‐SiO_2_‐Mix) from construction to Point‐of‐care application. F) LAMP results under different reaction temperatures (60–65 °C). (G) Comparable DNA extraction performance with different lysis under room temperature (25 °C, green), 62 °C (blue), and sequential 56 °C to 70 °C (purple). (H) LAMP result of testing six *Vpa* samples simultaneously. Lanes 1‐6: six *Vpa* samples; PC: positive control using commercial‐kit‐extracted DNA. I) LAMP results with different reaction times. J) The detection results of *Vpa* with concentrations from 10^9^ to 10 CFU mL^−1^ for LOD determination. K) Specificity. *Vro*‐*V. rotiferianus*; *Vha‐V. harveyi*; *Vsp‐V. splendidus*; *Val‐V. alginolyticus*. L) Detecting *Vpa* from animal tissues; Lanes T1‐T3: three tissue samples. (J) Detecting *Vpa* from seawater; Lanes W1‐W3: three seawater samples. NC: negative control.

Previous work established the suitability of the S(PAA‐SiO_2_‐Mix) device for rapid NA extraction and PCR‐based diagnosis in bacteria, such as *Vpa*. To further validate the device's applicability, DNA was extracted using S(PAA‐SiO_2_‐Mix) from diseased *Antheraea pernyi* (*A. pernyi*) pupae infected with *nucleopolyhedrovirus (NPV*, a viral pathogen of *lepidopteron)* and *Nosema pernyi* (*N. pernyi*, *Np*, a parasite cause pébrine disease) (*n*  =  3 per group). Conventional PCR successfully amplified target gene fragments of 151 and 173 bp (**Figure** **6**A,B). Concurrently, mold collected from the surface of decaying fruit was subjected to PCR using universal fungal primers (NS‐1 and NS‐4) target 18SrRNA of the fungus, yielding a product of ≈1.1 kb (Figure 6C). The performance was comparable to DNA extracted using a commercial kit. These results demonstrate the broad application potential of the S(PAA‐SiO_2_‐Mix) device as a functional unit for template preparation in molecular pathogen detection.

### 2.6 Field‐Deployable Diagnosis of *Vibrio parahaemolyticus*

#### 2.6.1 Field‐Deployable Diagnosis Process

*Vpa*, a well‐documented foodborne pathogen transmitted through the food chain,^[^ ^33^ ^]^ is responsible for severe gastrointestinal diseases in humans.^[^ ^34^ ^]^ This pathogen is prevalent in aquaculture species and can be transmitted to humans through improperly processed seafood, causing acute food poisoning.^[^ ^35^ ^]^ Hence, we isolated the pathogen from the infected sea cucumbers and employed the 16S rRNA gene as target gene for LAMP coupled with DNA colorimetric detection. By integrating the rapid and straightforward fabrication of device S(PAA‐SiO_2_‐Mix) with an efficient DNA extraction process, we established a comprehensive rapid visual detection system for *Vpa*, spanning from raw material processing to point‐of‐care pathogen testing, as illustrated in Figure 6E.

Figure 6E illustrates the minimal equipment requirements of our complete technical process. The fabrication of the S(PAA‐SiO_2_‐Mix) device, based on photopolymerization principles, requires only a mini UV lamp. Unlike conventional PCR with its stringent temperature cycling requirements, LAMP amplification eliminates the need for precise temperature control equipment, utilizing instead a simple constant temperature device. We developed a LAMP detection system featuring two temperature zones: a reaction zone for isothermal amplification and a result zone for enzyme inactivation, enabling more accurate colorimetric result interpretation. The entire system is portable and can be powered by mobile energy sources, making it ideal for field applications.

#### 2.6.2 Visualized LAMP and Simplified Lysis

We systematically optimized the LAMP reaction parameters by evaluating temperature gradients (60–65 °C in 1 °C increments; Figure 6F) and reaction durations (10–30 min at 5‐min intervals; Figure 6I). Successful target gene amplification occurred across all tested temperatures, with 62 °C selected as optimal temperature for the reaction zone due to its tolerance for minor fluctuations (±2 °C). Further experiments established 20 min as the optimal LAMP reaction duration. Amplification was near‐complete within 15 min, permitting accurate visual results under natural light. However, UV illumination revealed a potential false‐positive risk. The visual strategy using a hybrid dye system (hydroxynaphthol blue (HNB) and calcium phthalocyanine) enhanced color resolution under ambient light while providing complementary verification under UV light, thereby improving overall accuracy.

Considering that the optimal working temperature for Proteinase K in biological sample lysis typically ranges from 55–60 °C, we tested enzyme lysis at both room temperature (15–25 °C) and a constant temperature of 62 °C to reduce equipment dependency in resource‐limited settings. As shown in Figure 6G, extractions with simplified single lysis step under room temperature or 62 °C finally yielded slightly lower DNA quantities (22.5–32.5 and 28.95–35.76 ng µL^−1^, respectively) compared to that with standard lysis conditions (sequential heating at 56 °C and 70 °C, 48.11–57.63 ng µL^−1^). Nevertheless, these methods with simplified lysis produced comparable quality that met the requirements for subsequent PCR experiments (Figure 6D). Besides, room temperature lysis enables equipment‐free nucleic acid (NA) extraction in field settings. To address potential efficiency reductions in colder environments, on‐demand heating to 62 °C can be implemented using the same portable module before the subsequent LAMP reaction, further minimizing equipment requirements.

#### 2.6.3 Sensitivity, Specificity, and Detection Range

Field‐deployable diagnosis for *Vpa* (FD‐*Vpa* for short) integrated room temperature lysis, DNA fast extraction via S(PAA‐SiO_2_‐Mix), and visual LAMP testing was established. The entire process is highly time‐efficient and labor‐saving. Preparation of S(PAA‐SiO_2_‐Mix) devices and NA extraction can be completed rapidly, with the entire process requiring as little as 50 min. The most time‐consuming steps ‐enzymatic lysis (10‐15 min) and LAMP amplification (20 min) ‐ require low labor. When using pre‐fabricated S(PAA‐SiO_2_‐Mix) devices, the workflow from sample collection to result interpretation is reduced to under 40 min, demonstrating significant advantages for rapid *Vpa* detection. Concurrent detection of six *Vpa* samples and a positive control (using commercial‐kit‐extracted DNA) confirmed high‐throughput capability (Figure 6H). The number of simultaneous detections can be flexibly scaled to meet field requirements (e.g., 15, 95, etc.) through modular expansion via adjustable connection zones.

The limit of detection (LOD) accessible by FD‐*Vpa* was determined using serially diluted *Vpa* cultures. As shown in Figure 6J, the LOD for the FD‐*Vpa* assay reached as low as 10 CFU mL^−1^, consistent with previously reported values,^[^ ^36^ ^]^ demonstrating its high sensitivity. Other *Vibrio* species (*V. rotiferianus*, *V. harveyi, V. splendidus*, and *V. alginolyticus*), isolated from the same diseased sea cucumber and maintained in the laboratory, were used to evaluate specificity. As shown in Figure 6K, the FD‐*Vpa* assay yielded negative results for all four other *Vibrio* species but positive result for *Vpa*, indicating its high specificity.

As aquatic products are a primary transmission route for *Vpa*, pathogen detection in the products themselves and their aquaculture environment (seawater) is crucial for timely warnings and implementing control measures. Therefore, we applied the FD‐*Vpa* method to detect *Vpa* in the body wall of *Vpa‐*infected sea cucumbers and in surrounding seawater samples. Successful visual detection of *Vpa* was achieved in both sample types (Figure 6L,M).

The FD‐*Vpa* detection system is designed for field‐deployable detection with minimal equipment, lower cost, and simplified rapid workflows. Compared to other reported sensitive on‐site point‐of‐care methods (LOD: 1‐430 CFU mL^−1^) over the past decade, FD‐*Vpa* maintains competitive sensitivity (LOD: 10 CFU mL^−1^) while demonstrating comprehensive advantages (**Table** **1**). Key advantages include: 1) Minimal equipment ‐ requires only a portable heater for LAMP amplification, eliminating additional devices such as centrifuges (for DNA extraction),^[^ ^37^ ^]^ turbidimeters,^[^ ^38^ ^]^ rotary systems (for microfluidic methods),^[^ ^39^ ^]^ or potentiostats;^[^ ^40^ ^]^ 2) Rapid processing ‐ achieves the shortest end‐to‐end “sample‐to‐result” time (40 min);^[^ ^41^ ^]^ 3) Scalable throughput: Flexibly accommodates high‐throughput workflows (e.g., 95 samples treated simultaneously on a 96‐well plate), surpassing reported low^[^ ^37^, ^38^, ^40^, ^41^, ^42^ ^]^ or limited‐throughput^[^ ^39^ ^]^ (typically ≤18 samples) methods; 4) Reduced contamination risk ‐ compared to heat‐based lysis,^[^ ^40^, ^41^, ^42^ ^]^ room‐temperature lysis lowers aerosol contamination during multi‐sample processing, which is critical for result accuracy; 5) Reusable samples ‐ open DNA extraction using S(PAA‐SiO_2_‐Mix) preserves sample DNA for downstream analyses, unlike single‐use consumables in integrated systems; 6) Cost‐effectiveness ‐ The S(PAA‐SiO2‐Mix) substrate offers unparalleled affordability versus expensive microfluidic chips^[^ ^39^ ^]^ or gold nanoparticles.^[^ ^42^ ^]^ This integrated FD‐*Vpa* system provides a promising approach for on‐site pathogen detection in resource‐limited settings.

**Table 1:** Comparisons of different point‐of‐care LAMP detection methods for *Vpa*.

Method	DNA template preparation methods	Necessary equipment	Detection time^a)^	LOD	Refs.
FD‐*Vpa* detection	S(PAA‐SiO_2_‐Mix)‐based fast, high‐throughput DNA extraction	A portable heater.	40 min (LAMP: 20 min)	10 CFU mL^−1^	This study
HRP‐mimicking molecular beacon‐based colorimetric LAMP	Commercial kit.	Heater; Centrifuge	60 min (LAMP: 30 min)	1 CFU mL^−1^ in buffer; 10 CFU g^−1^ in fish sample	[37a]
Probe‐based LAMP on paper membrane	Commercial kit.	Portable heater; Centrifuge; Fluorescence microscope	Not Provided (LAMP: 30 min)	10^2^ copies per reaction	[37b]
Nephelometric analysis LAMP	No detailed description provided.	Heater; LoopAmp real‐time turbidimeter.	Not Provided (LAMP: 60 min)	1 CFU/reaction (10^2^ CFU g^−1^)	[38]
Hydrogel‐based EC‐LAMP micro‐chip	Commercial kit.	Heater; Rotator; Centrifuge.	68 min (LAMP: 15 min)	2.11 copies/reaction	[39a]
Integrated rotary microfluidic LAMP	Bead‐based DNA extraction.	A rotary system consists of (1) three heating blocks, (2)a servo motor, and (3) a CCD camera.	80 min (LAMP: 60 min)	50 CFU mL^−1^	[39b]
Centrifugal microfluidic LAMP	Bead‐based DNA extraction.	Heater; Rotator;	65 min (LAMP: 15 min)	10^2^ cells mL^−1^	[39c]
LAMP on integrated centrifugal microdevice	Bead‐based DNA extraction.	Portable genetic analyzer integrated (1) a spindle motor, (2) a couple of Minco heaters, and (3) a UV–visible optical detector.	65 min (LAMP: 60 min)	10^2^ cells mL^−1^	[39d]
Graphene‐based screen‐printed electrochemical LAMP	Suspended (NaOH), boiled (95 °C, 5 min), neutralized, and centrifuged (5 min). Supernatant was used as DNA template.	Portable heater; mini‐potentiostat	≈50 min (DNA extraction 10 min +LAMP 40 min)	8 CFU mL^−1^; 0.08CFU/g	[40]
Poly‐L‐lysine functionalized silica membrane‐enhanced colorimetric LAMP (pLAMP)	Heated (95 °C, 5 min); captured by PL‐SM and purified; PL‐SM‐DNA complexes used as template.	CFX Connect™ Real‐Time PCR System	50 min (LAMP: 30‐60 min)	1 CFU mL^−1^	[41]
Gold nanoparticle‐based LAMP	Lysis (boiled water, 10 min), chilled in ice bath (10 min), and centrifuged (5 min). Supernatant was used as DNA template.	Heater; Centrifuge	≈60 min (DNA extraction 25 min + LAMP: 30 min + result reading: 5min)	8.6 CFU/reaction (430 CFU mL^−1^)	[42]

^a)^ From sample to results, including DNA template preparation and results analysis.

## 3 Conclusion

In this study, an innovative DNA extraction functional device S(PAA‐SiO_2_) based on a hydrogel matrix was developed through an acrylic acid‐free radical polymerization reaction. This novel approach offers several significant advantages: simple preparation requiring no specialized technical skills, rapid fabrication within 5–10 min, cost‐effectiveness at ≈$0.028 per unit, high‐throughput production capacity (96/384 units per batch), and minimal equipment dependency (only requiring a compact UV lamp measuring 15 cm × 8 cm × 6 cm). These features make it particularly suitable for implementation in low‐resource settings.

Furthermore, the modification of SiO_2_ particles on the surface and their impacts on the properties of interface DNA adsorption were investigated in detail. The results revealed that SiO_2_ modification maintains structural thermal stability, enhances material rigidity, and the surface properties, such as roughness, specific surface area, and interface hydrophobicity, and simultaneously reducing surface electronegativity. The device operates through a multimodal DNA adsorption mechanism, primarily governed by salt‐bridge interactions and hydrophobic forces. The S(PAA‐SiO_2_) devices demonstrate superior performance due to their optimized surface characteristics, exhibiting DNA extraction yields comparable to commercial kits and significantly higher than the non‐SiO_2_‐modified S(PAA) device. The performance hierarchy follows: S(PAA‐SiO_2_‐Mix) > S(PAA‐SiO_2_‐Max) > S(PAA‐SiO_2_‐Mini) > S(PAA). Notably, the S(PAA‐SiO_2_‐Mix) device demonstrates efficient and fast DNA elution, with most DNA recovered during the first elution. Furthermore, the device can be reused and maintains structural integrity through three reuse cycles, demonstrating excellent reusability.

Results showed the separator's function as a core component for DNA extraction in molecular diagnostics, demonstrating its potential value for detecting bacterial (*Vibro*), viral (*Npv*), parasitic (*Np*), and fungal pathogens. By integrating the rapid fabrication of S(PAA‐SiO_2_‐Mix), high‐throughput DNA extraction, and visual LAMP detection, we established a comprehensive rapid detection system for *Vpa*, FD‐*Vpa*, spanning from raw material processing to point‐of‐care clinical testing. This integrated technology offers the key advantages of simple operation, rapid processing (within 40 min), sensitive (LOD: 10 CFU mL^−1^), specific and accurate (hybrid dye system‐enhanced complementary verification) results. This system shows tremendous potential for pathogen diagnostics in outdoor and resource‐limited settings. Our work provides significant contributions to the field of rationally designed bioengineering separation devices with optimized surface properties and rapid pathogen detection systems tailored for low‐resource environments, offering new directions for future research in point‐of‐care diagnostics.

## 4 Experimental Section

### Reagents and Materials

All chemical reagents utilized for the fabrication of DNA extraction devices were of analytical grade and were used without further purification. Deionized water was employed throughout all experimental procedures. The following chemicals were procured from Aladdin Biochemical Technology Co., Ltd. (Shanghai, China): acrylic acid (AA, 99%), PEGDA(Mn = 400), and (2,4,6‐trimethylbenzoyl)diphenylphosphine oxide (TPO, 98%). Essential reagents, including ethylenediaminetetraacetic acid (EDTA, 99%), Tris‐HCl (pH 8.0, 99%), copper sulfate pentahydrate (CuSO_4_·5H_2_O, 99%), and sodium chloride (NaCl, 99%), were obtained from Tianli Chemical Reagents Co., Ltd. (Tianjin, China).

SiO_2_ quartz sands with varying particle sizes (mini: 40–100 µm, max: 180–270 µm) were purchased from Aladdin Biochemical Technology Co., Ltd. (Shanghai, China). Hydrogen peroxide (H_2_O_2_, 30% w/w) and anhydrous ethanol (99.7%) were supplied by Kemeo Chemical Reagents Co. (Tianjin, China). Dopamine hydrochloride (98%) and polyethylene glycol (PEG, Mw = 400) were acquired from Aladdin Reagents Co. (Shanghai, China).

For the DNA extraction process, sodium dodecyl sulfate (SDS, 99%) and Proteinase K (≥30 units mg^−1^ protein) were obtained from Sangon Biotechnology Co., Ltd. (Shanghai, China). Commercial column‐based DNA extraction kits (Animal Tissues/Cells Genomic DNA Extraction Kit) were provided by Solarbio Technology Co., Ltd. (Beijing, China) for comparative studies. Bacterial samples used for DNA extraction studies consisted of *Vibrio* strains isolated from aquatic products maintained in our laboratory.

The primers used in conventional PCR and LAMP processes were synthesized by Sangon Biotechnology (Shanghai) Co., Ltd. (Shanghai, China). Enzymes and buffers, including LA Taq, 10× LA PCR Buffer II (Mg^2+^ Plus), dNTP Mixture (each 2.5 mm), BcaBEST polymerase, and 2× BcaBEST buffer, were purchased from TAKARA Biomedical Technology (Dalian) Co., Ltd. (Japan).

### Preparation of S(PAA) Devices

The S(PAA) devices were fabricated using commercial 0.2 mL centrifuge tubes as molds. A prepolymer solution composed of 49.99 wt.% AA, 49.99 wt.% PEGDA, and 0.02 wt.% TPO was prepared and dispensed into each mold to 2/3 height, approximately. A toothpick or bamboo stick was vertically inserted into the solution to serve as a handling grip. Photo‐induced free radical polymerization was initiated by exposing the molds to UV light (280–400 nm wavelength) for 3–10 min using a portable UV lamp (15 cm × 8 cm × 6 cm). The devices were released from the mold through the handle. To ensure dimensional uniformity, the polymerized functional segments of all devices were trimmed to a standardized height of 10 mm. Finally, the devices underwent sequential washing cycles with deionized water and anhydrous ethanol and were air‐dried for subsequent experiments.

### Preparation of S(PAA‐SiO_2_) Devices

For S(PAA‐SiO_2_) device preparation, the base prepolymer solution (49.99 wt.% AA, 49.99 wt.% PEGDA, 0.02 wt.% TPO) was homogenized with SiO_2_ particles at a 1:1 mass ratio. Three SiO_2_ particle sizes were employed: 1) mini (40–100 µm), 2) max (180–270 µm), and 3) mix (ratio_(mini:max)_ = 1:1). The particle‐loaded solutions were thoroughly mixed. The composite mixtures were then cast into 0.2 mL centrifuge tube molds and photopolymerized under UV irradiation (280–400 nm) for 3–10 min. The devices were released from the mold through the handle. All devices were trimmed to a consistent functional segment height of 10 mm. Residual monomers and unbound particles were removed through sequential washing in deionized water and anhydrous ethanol. Finally, the devices were air‐dried for further use.

### Assemble High‐Throughput DNA Separators

The comb‐shaped array device is composed of 8 individual devices assembled side by side on the connecting arm. The fabrication process was as follows: Ultra‐light clay was shaped according to the length of an eight‐channel strip, forming a strip 8 cm × 1 cm × 0.4 cm in size. The strip was left at 25 °C for 10 min, and then holes were made at 1 cm intervals using a toothpick, with a total of 8 holes. The strip was left at 25 °C for 2 h to set.

### DNA Extraction via Separators

*Vibrio* strains were cultured in commercial 2216E medium (peptone 5.0 g L^−1^, yeast extract 1.0 g L^−1^, ferric citrate 0.1 g L^−1^, sodium chloride 19.45 g L^−1^, magnesium chloride 5.98 g L^−1^, sodium sulfate 3.24 g L^−1^, calcium chloride 1.8 g L^−1^, agar powder 15 g L^−1^) at 28 °C for 14 h, then centrifuged to remove the supernatant, and the bacterial pellets were stored at ‐20 °C for later use.

For comparison with commercial DNA extraction Kits, the same lysis system was used. In a 2.0 mL centrifuge tube, the bacterial sample (∼0.4 mg per 100 µL lysis buffer) was added, along with 200 µL of GA Buffer (Solarbio® Life Sciences, DP324) and 20 µL of Proteinase K (20 mg mL^−1^). The tube was incubated at 56 °C for 10–15 min. Then, 200 µL of GB Buffer (Solarbio Life Sciences, DP324) was added and heated at 70 °C for 10 min to obtain the NA‐containing lysate. For low‐resource setting applications, the following simplified lysis process was used: the bacterial sample was added to a centrifuge tube containing 100 µL lysis buffer (100 mm of Tris‐HCl, 25 mm of EDTA, 500 mm of NaCl, 1 wt.% SDS, and 10 µg mL^−1^ proteinase K), and incubated at room temperature or 62 °C for 10–15 min.

The recovery of PCR product involved the following steps: The target band was excised under UV illumination using a scalpel. Gel Lysis Buffer (Sangon Biotech, Buffer B2 in SanPrep Column DNA Gel Extraction Kit, Lot: 1224 KA3702) was added at a ratio of 400 µL per 100 mg of gel slice. The mixture was incubated at 50 °C for 10 min to dissolve the gel. Then, DNA fragment was extracted using S(PAA‐SiO_2_‐Mix).

For the DNA extraction process, the volume of the functional buffers was determined maximum solution processing capacity, and higher nucleic acid extraction yield. The total volume of the binding matrix was set at 160 µL (a volume of 170–180 µL requires extremely careful agitation when using a 0.2 mL tube), while the elution was set at 80 mL, which is sufficient to ensure that the functional area is fully immersed, thereby achieving effective elution. Therefore, the process applicable to the most common molecular biology consumables (2.0 mL / 0.2 mL tubes) was: 100 µL of the lysate was mixed with 60 µL of isopropanol in a 0.2 mL centrifuge tube. The device was inserted into the tube, shaken up and down for 0.5–1 min, then removed. A new 0.2 mL centrifuge tube was added with 150 µL of 75% (v/v) ethanol, and the device was shaken gently for 30 s. Afterward, the device was removed and air‐dried at room temperature for ≈5 min. Finally, a new 0.2 mL centrifuge tube with 80 µL of 1×TE buffer (10 mmol L^−1^ Tris‐HCl; 1 mmol L^−1^ EDTA; pH 7.8–8.2) was added, and the device was shaken for 1 min to elute the DNAs. To obtain a total elution yield, the elution step was repeated another two times. The extracted DNA was used immediately or stored at ‐20 °C.

*Vpa* cells were suspended in seawater to a final concentration of ≈10^3^ CFU mL^−1^ for seawater sample preparation. A 4× concentrated lysis buffer was added to the seawater sample at a volume ratio of 3:1 (seawater: lysis buffer). The mixture was incubated at room temperature for 10 min. DNA was subsequently extracted following the procedure described above.

Hundred microliters of *Vpa* suspension was injected into the body wall of the sea cucumber for tissue sample preparation. The tissue blocks were cut and homogenized. 1× lysis buffer was added to the homogenized tissue. The mixture was then incubated at room temperature for 30 min. DNA was subsequently extracted following the procedure described above.

### DNA Extraction Using Column‐Based Commercial Kit

Genomic DNA extraction was performed using the Marine Animal Tissue Genomic DNA Extraction Kit (Solarbio® Life Sciences, DP324), following the manufacturer's protocol. Briefly, the bacterial culture medium was pelleted by centrifugation (1 min, 12000 rpm) and resuspended in 200 µL GA buffer via 15‐s vortexing. Subsequently, 20 µL Proteinase K (20 mg mL^−1^) was added, mixed thoroughly, and briefly centrifuged to remove condensate from the tube lid. The mixture was incubated at 56 °C until complete tissue lysis (10–30 min), followed by brief centrifugation to eliminate residual condensate. Next, 200 µL GB buffer was added, inverted 10 times for homogenization, and incubated at 70 °C for 10 min, yielding a clear lysate. After centrifugation, 200 µL of anhydrous ethanol was added, then the lysate was transferred to a spin column. The column was centrifuged (12000 rpm, 30 s), and the flow‐through was discarded. Then, the column was washed sequentially with 500 µL GD buffer and 600 µL PW wash buffer (each step followed by centrifugation at 12000 rpm for 30 s). After discarding the flow‐through, the column was transferred to a fresh collection tube and centrifuged (12000 rpm, 2 min) to remove residual ethanol. The membrane was air‐dried at room temperature for 5 min. Finally, DNA was eluted using 100 µL TE buffer from the column membrane, incubating for 2–5 min at room temperature, and centrifuging (12000 rpm, 2 min). To increase the total DNA yield, the eluate was reloaded onto the column membrane, and the elution step was repeated another 1‐2 times. The final eluate was collected for downstream applications or stored at ‐20 °C.

### Reuse of Device S(PAA‐SiO_2_)

After DNA extraction, the device S(PAA‐SiO_2_) was washed with water five times. The functional area of the device was immersed in 150–1000 µL of water, shaken up and down for 1–3 min, then removed and centrifuged to remove excess water before being transferred to a new volume of water for the next wash. The cleaned devices were air‐dried before being reused.

### Characterization

Surface morphology was analyzed via optical microscopy (Olympus BX53) and field‐emission scanning electron microscopy (FE‐SEM, Zeiss Sigma 300). Surface roughness was quantified using a 3D confocal microscope (Leica DCM8). CA measurements were performed with a goniometer (JC2000C, Powereach) to assess solution wettability on the devices. Chemical composition was determined by FTIR (Nicolet iS50), and thermal stability was evaluated via TGA (TA Instruments Q500). The viscosity‐elasticity response characteristics of the devices were determined using the strain‐controlled rheometer Anton Paar 302e through the amplitude scanning mode.

The viscoelastic properties of the materials were determined using an Anton Paar MCR 302e strain‐controlled rheometer in amplitude sweep mode. Of Mechanical Testing, the initial modulus and tensile failure properties of the samples were evaluated using a universal testing machine. Dumbbell‐shaped hydrogel specimens with a gauge dimension of 20 × 10 × 3 mm (length × width × thickness) were subjected to uniaxial tensile testing at room temperature until fracture, employing a crosshead speed of 2 mm/min. Force‐displacement data were recorded. All four materials exhibited characteristic nonlinear force‐displacement curves indicative of viscoelastic behavior. Elastic modulus was calculated as follows: 1) Engineering stress σ = F / S_0_ (F‐applied force, S_0_‐the original cross‐sectional area). 2) Engineering strain $\varepsilon =\frac{\Delta L}{L}$ 3) Young's modulus $E=\frac{\sigma }{\varepsilon }$. 4) Toughness T = $\int_{\varepsilon_{0}}^{\varepsilon_{f}}\sigma \left(\varepsilon \right)d\varepsilon$.

DNA concentration and purity were measured using a UV–vis spectrophotometer (NanoUV‐300, Thermo Fisher Scientific). Absorbance ratios A260/280 and A260/230 were used to evaluate potential protein, organic solvent, and salt contamination.

### Conventional PCR Diagnosis

In each 25 µL PCR reaction mixture, the following components were included: 1.25 U TaKaRa LA Taq, 2.5 µL 10× LA PCR Buffer II (Mg^2+^ Plus), 2.0 µL dNTP Mixture (each 2.5 mm), 0.5 µL of each primer, and 2 µL DNA. PCR amplification was performed as follows: denaturation at 94 °C for 5 min; 35 cycles of denaturation (94 °C) for 30 s, annealing (60 °C for *Vpa* and *Np*, 65 °C for *Npv*, and 50 °C for fungus) for 30 s, and extension (72 °C) for 90 s; followed by a final extension at 72 °C for 5 min. The PCR products were separated in a 1% agarose gel and visualized using a BIO‐RAD ChemiDoc Imaging System. Used primers are shown in **Table** **2**.

**Table 2:** Sequence information of the primers.

Method	Target pathogen	Primer	Sequence (5′→3′)	Product size
Conventional PCR	*Vpa*	B‐27F	AGAGTTTGATCCTGGCTCAG	≈1.8 kb
		B‐1492R	TACGGCTACCTTGTTACGACTT	
	*Np*	16S‐F	GACGGAAGAATACCACAAGGAGT	173 bp
		16S‐R	CTATATGAGGGTCTCACATCTTGT	
	*Npv*	gp50‐F	GCGTCGACGGATCCAGTTCGTTATGTACGTGCGCTGG	151 bp
		gp50‐R	GCGACGTCGGTACCTCATTGCAGTGGTCCTGGTATTGG	
	Fungus	18S‐NS1	GTAGTCATATGCTTGTCTC	≈1.1 kb
		18S‐NS4	CTTCCGTCAATTCCTTTAAG	
LAMP	*Vpa*	F3	CGCTGACAATCGCTTCTCAT	
		B3	GTTCTTCGCTTTGGCAATGT	
		FIP	CTGTCACCGAGTGCAACCACTTAACCACACGATCTGGAGCA	
		BIP	GCATCACAATGGCGCTTCCCACCGTTGGAGAAGTGACCTA	
		LF	TGTTGATTTGATCTGGCTGC	
		LB	TAACCCGAACAGCTGGTTC	

### Visual LAMP

LAMP primers^[^ ^43^ ^]^ (**Table** **2**) were synthesized by Sangon Biotechnology (Shanghai) Co., Ltd. The LAMP reaction mixture, 25 µL in total, contained: 1 µL BcaBEST Polymerase, 12.5 µL 2× BcaBEST Buffer (TAKARA BcaBEST DNA Polymerase ver. 2.0), 0.4 µL EIP (100 pm) and BIP (100 pm), 0.1 µL F3 (10 pm) and B3 (10 pm), 0.2 µL LB/LF (10 pm), 0.25 µL Fluorescent Dye (FD, loopamp, SLP221), 240 µm HNB(CAS No. 63451‐35‐4), and 2 µL DNA template. The reactions were performed under 60–65 °C for 10–30 min, followed by enzyme inactivation under 90 °C for 1–2 min. Under visible light, blue indicates a positive result, while purple indicates a negative result. Under UV light, green indicates a positive result, while pink indicates a negative result.

For LOD determination, the viable count of *Vpa* in overnight cultures was quantified via standard plate counting first. Serial ten‐fold dilutions (10^−1^–10^−8^) were prepared by transferring 0.5 mL aliquots into 4.5 mL of sterile 2216E medium, followed by 30‐s vortex mixing at each step. From dilutions 10^−5^–10^−8^, 50 µL aliquots were spread onto pre‐dried 2216E agar plates (1.5% w/v agar) using sterile plastic spreaders. After 10‐min absorption, plates were inverted and incubated aerobically at 28 °C overnight. Colonies (≈50–200 CFU per plate) were manually enumerated. Original culture density (CFU mL^−1^) was calculated as: CFU mL^−1^ = (mean colony count) / (volume plated in mL × dilution factor). Then, serial sterile seawater dilutions of the culture (10^9^, 10^4^, 10^3^, 500, 10^2^, 50, and 10 CFU mL^−1^) were processed DNA Extraction for LAMP analysis. High‐concentration samples (10^9^ and 10^4^ CFU mL^−1^): 1 mL centrifuged; pellets extracted with S(PAA‐SiO_2_‐Mix). For low‐concentration samples (≤ 10^3^ CFU mL^−1^), there is no visible sediment that can be seen with the naked eye after centrifugation. To prevent pellet loss, a modified protocol was used. Lysis buffer was prepared using the sample dilution replaced water, and 1 mL modified lysis was used for DNA extraction: After 10‐min room‐temperature incubation, 600 µL isopropanol was added; S(PAA‐SiO_2_‐Mix) was inserted and stirred mildly (1 min); subsequent steps followed standard procedures. Lastly, extracted DNA from gradient dilutions underwent LAMP amplification. Sterile water served as negative control (NC). The LOD was defined as the lowest *Vsp* concentration yielding positive LAMP amplification when NC remained negative.

## Conflict of Interest

The authors declare no conflict of interest.

## Supporting information



Supporting Information

## Data Availability

The data that support the findings of this study are available from the corresponding author upon reasonable request.
