# Supplementary material for: Material‐to‐Application Integration: Rapid Fabrication of Field‐Deployable Hydrogel‐SiO2 DNA Separator for Low‐Resource Point‐of‐Care Diagnostics
Source: Adv Sci (Weinh). 2025 Aug 28;12(43):e08580. doi: 10.1002/advs.202508580 (PMC12631843; doi:10.1002/advs.202508580)
Supplement: Supplementary file 1 — Supporting Information [file ADVS-12-e08580-s001.docx]

**Material-to-Application Integration: Rapid Fabrication of Field-Deployable Hydrogel-SiO₂ DNA Separator for Low-resource Point-of-Care Diagnostics**

Peipei Li^a^, Xinrong Li^a,b^, Haojie Wu^a,b^, Dongmei Yue^a^, Yubo Shang^a^, Shan Gao^a,c^, Bai Wang^a,c^, Xiaobin Jiang^b^^[[1]](#footnote-0)^*, Jingwei Jiang^a,c*^, Zunchun Zhou^a,c*^

*^a^ Ministry of Agriculture and Rural Affairs Key Laboratory of Aquatic Germplasm Resources Conservation and Utilization, Liaoning Ocean and* *Fisheries Science Research Institute,* *Liaoning Academy of Agricultural Sciences, Dalian,* *Liaoning, 116023, China*

*^b^ State Key Laboratory of Fine Chemicals,* *School of Chemical Engineering, Dalian University of Technology,* *Dalian, Liaoning, 116024, China*

*^c^ Liaoning Key Laboratory of Germplasm Improvement and Fine Seed Breeding for Marine Aquatic Animals, Dalian, Liaoning, 116023, China*


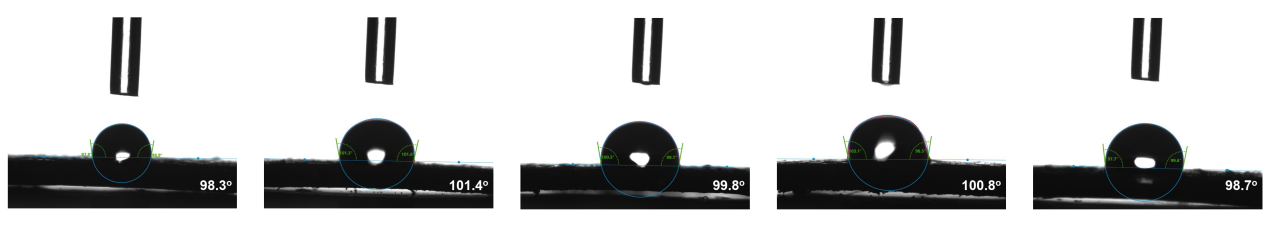


**Figure S1** Contact angle test of S(PAA-SiO₂-Mix) devices fabricated in five batches.

1. * Corresponding authors: E-mail: xbjiang@dlut.edu.cn(XB. Jiang), weijingjiang@live.cn (JW. Jiang), zunchunz@hotmail.com (ZC. Zhou). [↑](#footnote-ref-0)
